# The associations between suicide-related behaviors, prefrontal dysfunction in emotional cognition, and personality traits in mood disorders

**DOI:** 10.1038/s41598-022-22345-3

**Published:** 2022-10-17

**Authors:** Hisashi Kamimura, Takahiro Matsuoka, Hiroshi Okai, Naoki Shimizu, Shu Harada, Koji Matsuo

**Affiliations:** Department of Psychiatry, Faculty of Medicine, Saitama Medical University, 38 Morohongo, Moroyama, Iruma, Saitama 3500495 Japan

**Keywords:** Brain imaging, Bipolar disorder, Depression

## Abstract

Suicide is a serious public health problem, and it is urgent to identify biomarkers associated with suicide to prevent it. We aimed to clarify the association across suicidal behavior, personality traits, and brain activation by emotional stimulation tasks using near-infrared spectroscopy (NIRS) in patients with mood disorders. 11 mood disorder patients with a history of suicide attempt (MDSA), 18 mood disorder patients with no history of suicide attempt (MDNSA), and 17 healthy individuals were studied. The MDSA patients showed significantly high impulsivity and hopeless compared to healthy subjects, great response to the thread word task in the orbitofrontal cortex (OFC) and dorsolateral prefrontal cortex (DLPFC) compared to MDNSA patients, and the significant correlation between the personality traits and brain activation. The MDNSA did not show the trend. The results suggest that the personality traits and the activation of OFC and DLPFC during the negative emotional cognitive stimuli is associated with suicidal behavior, indicating the findings are involved in the pathophysiology of suicidality in mood disorders.

## Introduction

Suicide is a serious public health concern. According to the World Health Organization (WHO), more than 700,000 people worldwide die by suicide each year, and suicide was the fourth most common cause of death among those aged 15–29 in 2019^1^. Suicide is associated with mental illness, with depression being one of the most significant factors^2^. Patients with mood disorders, such as major depression disorder (MDD) and bipolar disorder (BD), have a higher risk of suicide than individuals with other psychiatric disorders^3^. Prevention of suicide are critical, and identification of biomarkers associated with suicide is warranted.

Suicidal behaviors may be associated with personality traits such as impulsivity, hopelessness, and pessimism^4,5^. Impulsivity is behaviorally defined as an impulse or behavior that occurs without considering the consequences of an action. Impulsive behavior is considered risky, and its consequences are often inappropriate^6^. Suicide-related studies have reported that among depressed patients with a history of abuse, suicide attempters are more impulsive than depressed patients without a history of abuse, and those who are more impulsive use more lethal means of suicide attempts^7,8^. Depression and hopelessness were associated with risk for suicide ideation and suicidal behaviors in substantial observational studies^9–13^. A meta-analysis of longitudinal studies was also reported that depression and hopelessness were associated with risk for suicide ideation, attempt and death^14^. A study examining the correlation between history of suicide attempts and personality traits in patients with major depression was found that hopelessness was a high risk factor for suicide attempts and that modifying hopelessness was important^15^.

The prefrontal regions are likely involved in the control of impulsivity and hopelessness^16,17^. In recent years, an increasing number of neuroimaging studies related to suicidal behaviors have been reported, and evidence of functional brain mechanisms has been accumulating. Mood disorders may impair emotion regulation^18^, including associations between high impulsivity and small orbitofrontal cortex (OFC) volume^19^. Studies using emotionally stimulating tasks in suicide research have found that strong serotonin 1a binding in the OFC and dorsolateral prefrontal cortex (DLPFC) was associated with suicide attempt lethality and the intensity of suicidal ideation^8^. A study of brain function in bipolar disorder showed a negative correlation between medial OFC response and hopelessness during negative emotional stimuli^17^. Studies measuring brain function in MDSA have reported an association between the frontal pole and right middle frontal gyrus and hopelessness^20,21^. Schmaal et al. reviewed 20 years of brain imaging studies on suicide and proposed a model of brain circuitry linked to suicide-related behavior (SRB) in which dorsal frontal regions (e.g., the DLPFC, the dorsomedial prefrontal cortex) and extensive ventral frontal regions (e.g., the OFC, ventral striatum, thalamus, cerebellum, dorsal anterior cingulate gyrus, and insular regions) may form a network and contribute to such behavior^22^. Based on these findings, we hypothesized that dysfunction in these brain regions is involved in SRB, and that these regions may also be associated with personality traits such as impulsivity, hopelessness, and depression.

In this study, we used near-infrared spectroscopy (NIRS) to measure brain function. NIRS is a technique that harnesses near-infrared light to gauge changes in the blood volume of oxygenated hemoglobin (Oxy-Hb) on the brain’s surface to investigate hemodynamics in the cerebral cortex. Diverse studies indicate that NIRS signals reflect cerebral hemodynamic responses^23,24^. The advantages of NIRS are that it is non-invasive and does not expose the brain to radiation, unlike brain imaging studies using single photon emission computed tomography (SPECT) or positron emission tomography (PET); the measurement time is in the order of 100 ms. It is useful for patients with psychiatric disorders who have difficulties remaining still for a long time—such as patients with agitated depression and attention-deficit hyperactivity disorder (ADHD)—because it has temporal resolution and does not require the participant to remain in the same position for long periods in a constrained environment, such as functional magnetic resonance imaging (fMRI). The most important point is that NIRS is an effective tool for assessing the quality of a patient’s condition. On the other hand, the disadvantages of NIRS are that measurement is limited to the brain’s surface and it is not possible to quantify deep regions (e.g., the anterior cingulate, hippocampus, amygdala) that play a role in mood regulation. Moreover, the spatial resolution is lower than that of fMRI. Few suicide-related studies on patients with mood disorders using NIRS have described an association between SRB and the frontal regions^20,25,26^. However, all of these studies incorporated verbal fluency tests (VFTs) to examine neuropsychological cognitive fluency. To the best of our knowledge, no studies have examined the relationship between personality traits and brain activation in response to emotional cognition, which is involved in the pathophysiology of mood disorders.

We aimed to establish whether frontotemporal activation during emotion recognition tasks differs between MDSA and MDNSA using NIRS, whether these differences are consistent with brain regions involved in suicide, as suggested by prior studies, and whether personality traits, such as impulsivity and hopelessness, are related to frontotemporal activation.

## Materials and methods

### Participants

We included 11 MDSA (seven with MDD; four with BD), 18 MDNSA (15 with MDD, three with BD), and 17 healthy individuals, statistically matched for age and sex. We recruited outpatients and inpatients at Department of Psychiatry, Saitama Medical University Hospital by advertisements and word-of-mouth communication; psychiatrists diagnosed them using a clinical interview according to the Diagnostic and Statistical Manual of Mental Disorders, 5th edition (DSM-5)^27^, as well as a structured interview using the Mini-International Neuropsychiatric Interview (M.I.N.I.)^28^. We included patients who met the diagnostic criteria for MDD and BD in the study. We excluded patients with comorbid personality disorders diagnosed through clinical diagnostic interviews. Healthy individuals were recruited by advertisements and word-of-mouth communication in the community. Among the healthy participants, we excluded those diagnosed with a psychiatric disorder through screening interviews using M.I.N.I., those who had a first-degree relative with a psychiatric disorder using a screening interview, and those who were taking psychotropic drugs continuously. Participants were also excluded if they had a history of head trauma with a loss of consciousness, organic brain disorder, neurological disease, alcohol or drug abuse or dependence, serious medical illness, or a first-degree relative with neuromuscular disease. We excluded left-handed participants from the dominant hand scale^29^. We assessed the estimated intelligence quotient (IQ) using the Japanese Adult Reading Test (JART)^30^, and we excluded those with an estimated IQ below 80. Mood symptoms using the 17-item Hamilton Depression Rating Scale (HAMD)^31^ for depressed symptoms and the Young Mania Rating Scale (YMRS)^32^ for manic symptoms were evaluated through interviews by trained psychiatrists. We employed the Columbia Suicide Severity Rating Scale (C-SSRS)^33^ to determine the severity of suicidal ideation, the number of suicide attempts, and the lethality of suicide attempts. We assessed impulsivity using the 11th edition^34^. The Barratt Impulsiveness Scale (BIS)^34^ is subclassified into attentional impulsiveness, motor impulsiveness, and non-planning impulsiveness, allowing for a detailed appraisal of impulsivity. In addition, we administered the self-report scale of the Quick Inventory of Depressive Symptomatology for Japanese (QIDS)^35^ to assess depression and the Beck Hopelessness Scale (BHS)^36^ to assess hopelessness. The study was approved by the Institutional Review Board of Saitama Medical University Hospital (Application No. 19038.03). This study was carried out in accordance with the latest version of the Declaration of Helsinki. All participants were fully informed of the study’s purpose in writing and orally, and those who gave written consent took part.

### NIRS measurement

**Figure 1:**
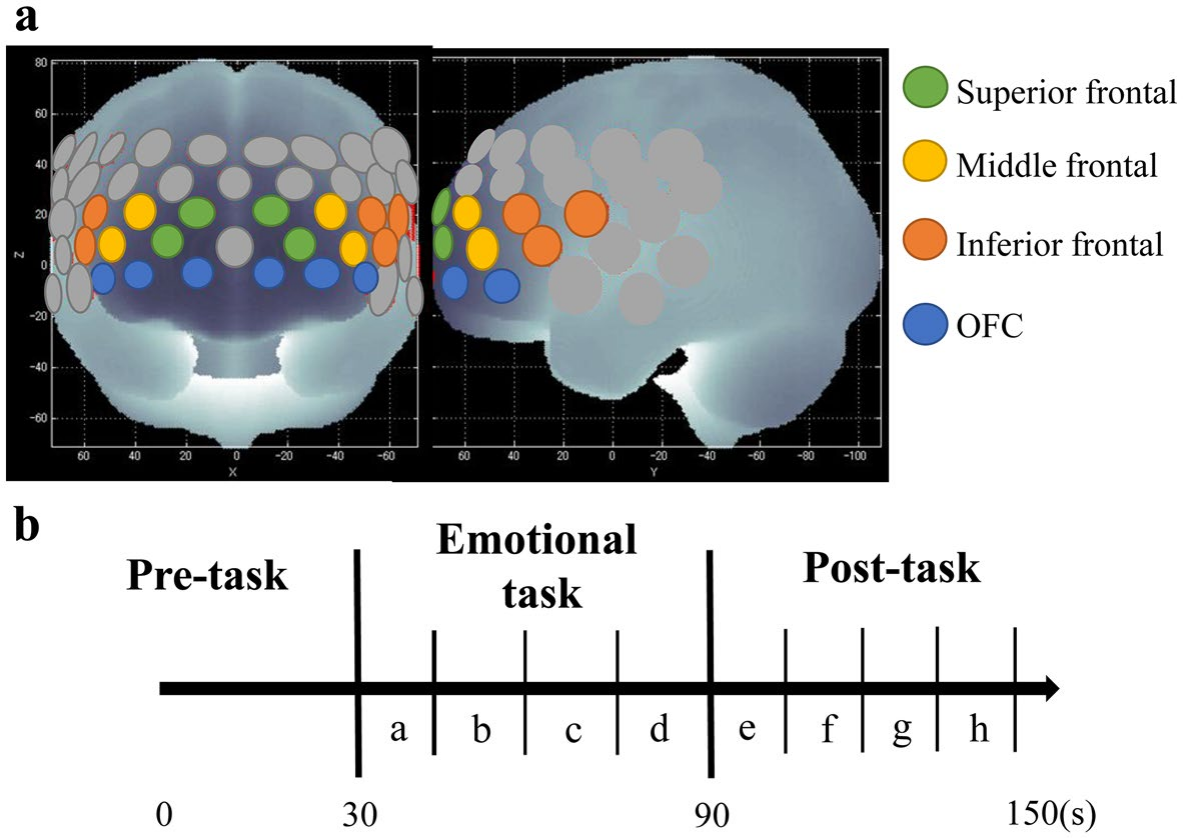
NIRS brain areas of measurement and periods of the emotional Stroop task. (**a**) Anatomical area measured in the NIRS. (**b**) Eight periods (a–h) within the task.

We used a 52-channel NIRS system (ETG-4100, Fujifilm Healthcare Corporation, Tokyo, Japan) with measurement probes arranged in a 3 × 11 pattern and symmetrically mounted around the frontotemporal area at a distance of 3 cm. The inferior channel was placed along T3-Fpz-T4 in the international 10–20 system of EEG. We identified the anatomical location of each channel using Montreal Neurological Institute coordinates^37^ (Fig. 1a). Based on the modified Beer-Lambert method, we harnessed two wavelengths of near-infrared light (695 nm and 830 nm) to track changes in Oxy-Hb concentration on the brain’s surface. We set the time resolution to 100 ms. We performed correction for artifacts due to body motion using a moving average method (moving average window: 5 s) and an algorithm to exclude channels showing noise and artifacts in accordance with previous studies^38,39^.

### The emotional cognitive stimulation task

We used the emotional Stroop task created by Matsubara et al.^40^ as the emotional cognitive stimulus task. The words were represented by two Chinese characters and belonged to one of the following categories: “happiness,” “sadness,” “threat,” or “neutral.” We used “happiness,” “sadness,” and “threat” for the target task and “neutral” for the control task. The words were displayed in red, blue, yellow, or green during NIRS measurements for 2 s per word using Presentation software (Neurobehavioral Systems, California, USA). “Happiness” has a positive emotional valence and moderate arousal (e.g., satisfaction, friendship), “sadness” has a negative emotional valence and low arousal (e.g., worry, impotence), “threat” has a negative emotional valence and high arousal (e.g., war, bankruptcy), and emotional stimuli cause more delayed responses than neutral stimuli^41,42^. The participants were instructed to press a button on the controller of the same color as the letter as quickly as possible while recalling the word. As a pre-task, 15 “neutral” words were shown for 30 s before the task. The task consisted of one of three emotion word segments—"happy,” “sad,” or “threat”—and a post-task emotionless word segment, each of which contained 30 words displayed for 60 s. The three types of emotional tasks were measured sequentially at one time, but the order of emotional tasks was presented in a pseudorandom order for each examinee to consider the effects of fatigue.

### Data analysis

#### Behavioral data

Behavioral indicators included the percentage of correct responses and reaction speed in each emotional task.

### NIRS data

We fitted Oxy-Hb data obtained through NIRS with a least-squares approximation using the average of the 10 s immediately before the task in the pre-task of the control task, and the average of the 5 s in the final segment of the control task after the task ended and was linearly fitted. To reduce the effects of inter-individual variability, we corrected for the standard deviation of the time variability of each individual’s change in Oxy-Hb. NIRS measurement units are in millimolar millimeters due to the inclusion of differential path-length factors^39^.

Only the lower three rows of the frontal area were used as channels for analysis based on previous NIRS studies^39,43^. The areas were divided into eight parts: the bilateral superior, middle, and inferior frontal regions, and the OFC region (Fig. 1a). In addition, to evaluate changes over time, we divided the measurement period into eight periods (four periods during the task, and four periods after the task) and calculated the mean Oxy-Hb for each period (Fig. 1b). We verified the validity of the number of interval settings using a power analysis. We set the power at 0.8, the significance level at 0.05, autocorrelation at 0.5, and the epsilon value at 0.75. We set the effect size at a medium effect of 0.25 based on Cohen’s guidelines for effect size^44^. We did so to check whether the sample size exceeded the total number of samples obtained.

### Statistical analysis

#### Demographic and behavioral data

To analyze the demographic data, clinical assessments, and behavioral data, we used χ^2^ tests for the categorical variables and ANOVA or *t*-tests for the continuous variables.

#### NIRS data

With respect to the number of period settings, the results of the power analysis indicate that the total number of samples required was at least 96 (each group: 4), which we deemed reasonable because the number of samples in the study exceeded this amount.

For Oxy-Hb, we performed ANOVA between two factors (diagnosis: three groups; brain areas: eight) and within one factor (time: eight periods) for each emotional cognitive stimulus. We also used an ANOVA defined by the OFC and superior frontal regions (including the DLPFC) from previous studies as the ROI^22^; with regard to the ROI, this entailed one between factor (diagnosis: three groups) and one within factor (time: eight periods). The power analysis shows that the total sample size required was at least 24 (each group: 8), and the number of samples in this study exceeded this quantity. We modified the degrees of freedom based on the work of Greenhouse–Geiser and we employed recalculated, significant probabilities. For the main effects and interactions that were substantially different, we performed ANOVA defined by one factor per period (diagnosis: three groups), followed by Sidak adjustment for multiple comparisons. We set the covariates for age and IQ. We utilized Pearson’s correlation coefficient to correlate Oxy-Hb with the clinical variables with respect to the period of the brain regions where these statistical analyses revealed considerable differences. We set statistical significance at p < 0.05 and performed statistical analyses using SPSS Statistics for Windows (version 27.0; IBM Corporation, Armonk, NY, USA).

## Results

### Demographic characteristics

There were no significant differences in sex, age, or the estimated intelligence quotient (IQ) among MDSA, MDNSA, and healthy individuals (Table 1). There were no significant differences between the MDSA and MDNSA groups in the proportion of patients with MDD and BD, number of mood episodes, with or without antidepressants, mood stabilizers, and psychotics. The intensity of suicidal ideation, assessed using the C-SSRS, were significantly higher in the MDSA group than in the MDNSA group. We observed considerable differences among the three groups using the HAMD, the QIDS, and the BHS, with MDSA and MDNSA patients scoring significantly higher than healthy individuals. For the BIS, we found significant differences among the three groups for the total score and motor impulsiveness, but not for attentive or non-planning impulsiveness. The total score and motor impulsiveness were significantly greater in MDSA than in healthy individuals, but we noted no considerable distinctions between healthy individuals and MDNSA, or between MDSA and MDNSA.

**Table 1:** Demographics, characteristics, and behavioral data of the participants.

	MDSA (n = 11)	MDNSA (n = 18)	Healthy (n = 17)	Statistics (F/χ^2^/t)	p-value	Post-hoc (p-value)
Age	49.0 ± 16.1	49.6 ± 14.5	52.8 ± 14.8	F = 0.29	0.751	–
Sex (male/female)	4/7	6/12	10/7	χ^2^ = 2.61	0.271	–
Diagnosis (MDD/BP)	7/4	15/3	n.a.	χ^2^ = 1.45	0.229	–
Age of onset	38.5 ± 16.3	40.4 ± 16.0	n.a.	t = 0.32	0.749	–
Duration (month)	138.2 ± 84.4	110.3 ± 116.9	n.a.	t = − 0.69	0.498	–
Estimated IQ	103.9 ± 10.1	103.3 ± 13.1	106.7 ± 9.7	F = 0.44	0.647	–
Hand dominance (score)	14 ± 0.8	14 ± 1.9	14 ± 0.7	F = 0.31	0.734	–
HAMD	9.1 ± 5.7	6.5 ± 5.3	0.0 ± 0.0	F = 17.29	< 0.001	MDSA > Healthy (< 0.001) MDNSA > Healthy (< 0.001)
YMRS	0.8 ± 1.0	0.7 ± 1.4	0.0 ± 0.0	F = 2.85	0.069	–
C-SSRS—Severity of suicidal ideation	4.6 ± 0.5	1.8 ± 1.9	0.0 ± 0.0	F = 44.14	< 0.001	MDSA > Healthy (< 0.001) MDSA > MDNSA (< 0.001) MDNSA > Healthy (< 0.001)
BIS total score	65.9 ± 16.0	61.9 ± 10.8	54.7 ± 7.3	F = 3.71	0.033	MDSA > Healthy (0.039)
BIS attentional impulsiveness	17.1 ± 5.8	16.1 ± 4.4	13.5 ± 2.8	F = 2.78	0.073	–
BIS motor impulsiveness	23.6 ± 5.6	20.9 ± 4.4	18.8 ± 2.4	F = 4.66	0.015	MDSA > Healthy (0.012)
BIS non-planning impulsiveness	25.2 ± 6.0	25.0 ± 5.3	22.5 ± 4.1	F = 1.38	0.263	–
QIDS	14.6 ± 5.1	10.6 ± 6.2	2.2 ± 3.0	F = 23.39	< 0.001	MDSA > Healthy (< 0.001) MDNSA > Healthy (< 0.001)
BHS	14.3 ± 3.8	11.3 ± 5.5	5.0 ± 1.9	F = 19.68	< 0.001	MDSA > Healthy (< 0.001) MDNSA > Healthy (< 0.001)
**Medication**						
Antidepressant (number; %)	10 (90.9)	15 (83.3)	n.a.	χ^2^ = 83.66	0.566	–
Mood stabilizer (number; %)	5 (45.5)	6 (33.3)	n.a.	χ^2^ = 33.66	0.514	–
Antipsychotic (number; %)	4 (36.4)	10 (55.6)	n.a.	χ^2^ = 55.66	0.316	–
**Behavioral data**						
Accuracy of happy (%)	96.4 ± 6.6	98.9 ± 2.0	99.4 ± 1.8	F = 2.62	0.084	–
Reaction time of happy (ms)	988.0 ± 22.4	904.8 ± 21.0	872.1 ± 23.1	F = 0.94	0.40	–
Accuracy of sad (%)	99.1 ± 2.2	98.9 ± 2.3	98.8 ± 2.6	F = 0.043	0.958	–
Reaction time of sad (ms)	962.0 ± 21.5	912.2 ± 19.5	865.1 ± 19.8	F = 0.786	0.462	–
Accuracy of threat (%)	97.9 ± 3.7	97.2 ± 5.3	97.8 ± 3.5	F = 0.117	0.889	–
Reaction time of threat (ms)	962.4 ± 19.5	934.5 ± 23.3	872.7 ± 18.2	F = 0.721	0.492	–

Mean ± SD, n (%), not applicable.

*MDSA* mood disorder patients with a history of suicide attempt, *MDNSA* mood disorder patients with no history of suicide attempt, *MDD* major depressive disorder, *BD* bipolar disorder, *HAMD* Hamilton Depression Rating Scale, *YMRS* Young Mania Rating Scale, *C-SSRS* Columbia Suicide Severity Rating Scale, *BIS* Barratt Impulsiveness Scale, *BHS* Beck Hopelessness Scale.

#### Behavioral data

There were no significant differences across MDSA, MDNSA, and healthy participants in the percentage of correct responses during the emotional Stroop task and task response reaction time (Table 1).

### NIRS data

Analysis of variance (ANOVA) defined between two factors (diagnosis: three groups; brain areas: eight) and within one factor (time: eight periods) in the entire brain area measured revealed no significant main effects of the brain region or period factors on any emotional task, and no significant interaction between diagnosis, brain region, and period. There was also no significant interaction between diagnosis and brain region, diagnosis and period, or brain region and period.

**Figure 2:**
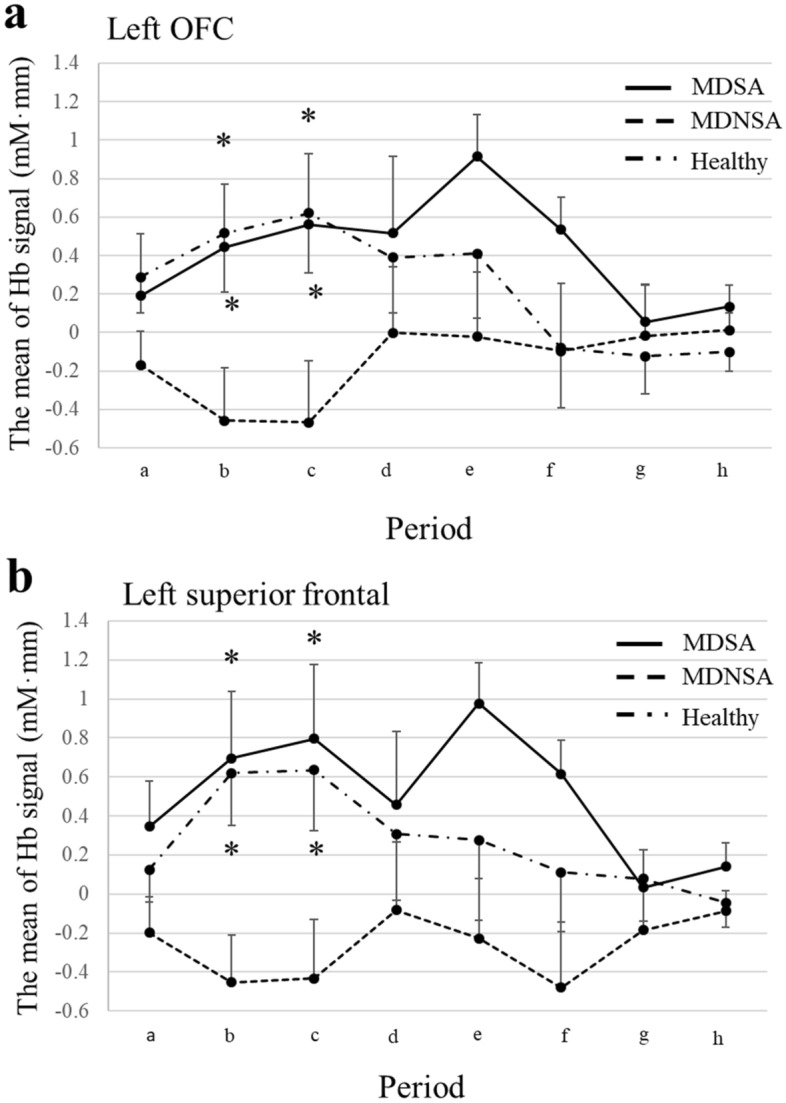
Mean changes of Oxy-Hb during the threat word task. (**a**) Significant differences between the MDSA and MDNSA groups, and between healthy participants and the MDNSA groups in periods b and c in the left OFC. *p < 0.05. (**b**) Significant differences between the MDSA and MDNSA groups, and between healthy participants and the MDNSA groups in periods b and c in left superior frontal area. *p < 0.05.

In the region of interest (ROI) analysis of the orbitofrontal areas, the left OFC area displayed significant differences on the threat word task; we noted a significant interaction between diagnosis and period (*F*(7.72, 158.22) = 2.23, *p* = 0.030, effect size(*η*_*p*_^2^) = 0.098), but no significant difference between the main effect of period and diagnosis. For the results, we analysis ANOVA within periods, ANOVA of diagnosis in the left OFC indicated significant differences between periods b and c (period b, *F*(2, 41) = 4.54, *p* = 0.017, *η*_*p*_^2^ = 0.181; period c, *F* (2, 41) = 5.38, *p* = 0.008, *η*_*p*_^2^ = 0.208) (Fig. 2a). As a post hoc analysis, in period b, MDNSA witnessed significantly smaller changes in the average concentration of Oxy-Hb than MDSA and healthy individuals (MDNSA vs. MDSA, p = 0.049; MDNSA vs. Healthy, p = 0.024). In period c, MDNSA experienced significantly smaller changes in Oxy-Hb than MDSA and healthy individuals (MDNSA vs. MDSA, p = 0.048; MDNSA vs. healthy, p = 0.020). There were no significant differences between MDSA and healthy individuals in either periods b or c.

Another ROI analysis of the left superior frontal area demonstrated significant findings during the threat word task; there was a significant main effect of diagnosis on the threat word task (F(2, 41) = 3.89, p = 0.028, *η*_*p*_^2^ = 0.160); however, there was no significant main effect of period or interaction between diagnosis and period. On the results main effect, we conducted a within-period ANOVA on diagnosis in the left superior frontal region. The results indicated significant differences in periods b and c (period b, F(2, 41) = 6.59, p = 0.003, *η*_*p*_^2^ = 0.243; period c, F(2, 41) = 4.96, p = 0.012, *η*_*p*_^2^ = 0.195). As a post hoc analysis, in period b, MDNSA had significantly smaller changes in Oxy-Hb than MDSA and healthy individuals (MDNSA vs. MDSA, p = 0.009; MDNSA vs. Healthy, p = 0.024). In period c, MDNSA underwent significantly smaller changes in Oxy-Hb than MDSA and healthy individuals (MDNSA vs. MDSA, p = 0.018; MDNSA vs. Healthy, p = 0.020) (Fig. 2b).

We utilized Pearson’s correlation coefficient between Oxy-Hb and the demographic data for periods b and c of the two ROIs on the threat word task, and we observed significant differences using ANOVA. In the left OFC, Oxy-Hb in period b was significantly and positively correlated with the BHS (*r* = 0.611, *p* = 0.046) and the QIDS (*r* = 0.704, *p* = 0.016) in MDSA. We found significant, positive correlations in the MDNSA between Oxy-Hb in the region and BIS motor impulsivity (*r* = 0.523, *p* = 0.026) in period b, and between Oxy-Hb and the total BIS score (*r* = 0.494, *p* = 0.037), attention impulsivity (*r* = 0.528, *p* = 0.024), and motor impulsivity (*r* = 0.629, *p* = 0.005) in period c.

**Figure 3:**
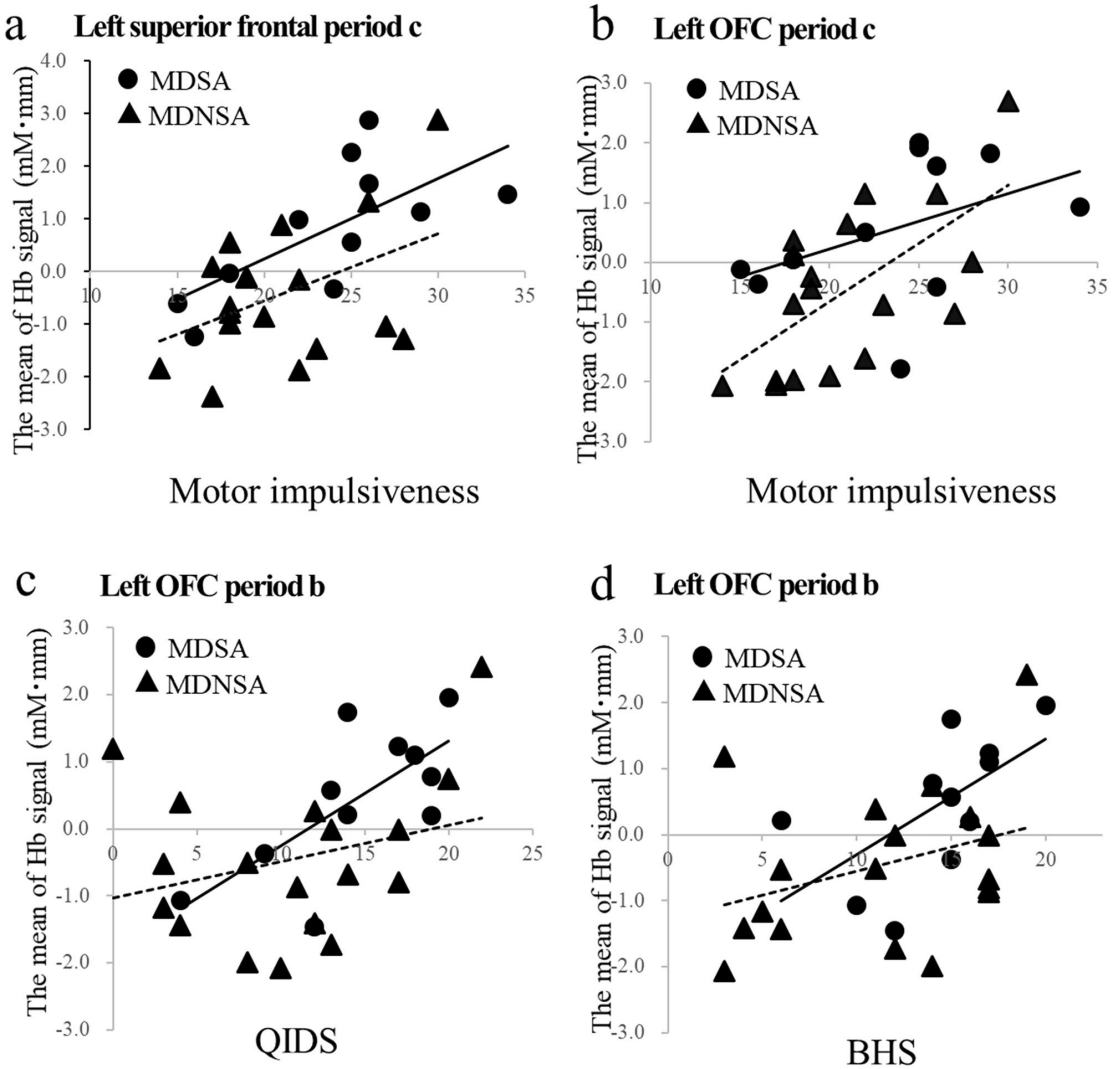
Correlation between brain activity during the threat word task and clinical demographics. Correlation between brain activity in period c and BIS motor impulsiveness in MDSA (r = 0.686, p = 0.020)—but not in MDNSA patients (r = 0.431, p = 0.074)—in the left superior frontal region (**a**) and in MDNSA patients (r = 0.629, p = 0.005)—but not in the MDSA group (r = 0.430, p = 0.187)—in the left OFC (**b**). Correlation of brain activity in period b of the left OFC region with QIDS in MDSA patients (r = 0.704, p = 0.016), but not in MDNSA patients (r = 0.290, p = 0.244) (**c**) and with BHS in MDSA patients (r = 0.611, p = 0.046) but not in MDNSA patients (r = 0.342, p = 0.164) (**d**). Solid line, regression line for the MDSA group; dotted line, regression line for the MDNSA group. *BIS* Barratt Impulsiveness Scale, *QUIDS* Quick Inventory of Depressive Symptomatology, *BHS* Beck Hopelessness Scale.

In the left superior frontal area, Oxy-Hb in period b was significantly and positively correlated with the QIDS score (*r* = 0.691, *p* = 0.018). Oxy-Hb in period c was significantly and positively correlated with the QIDS score (*r* = 0.674, *p* = 0.023) and BIS motor impulsivity (*r* = 0.686, *p* = 0.020) (Fig. 3) in MDSA. We observed significant and positive correlations in MDNSA for Oxy-Hb in period b and BIS attention (*r* = 0.480, *p* = 0.044) and motor impulsivity (*r* = 0.487, *p* = 0.040), and for Oxy-Hb in period c and BIS attention impulsivity (*r* = 0.601, *p* = 0.008). We did not find any substantial correlations with other demographic data.

## Discussion

In the current study, MDSA patients had greater activity in the DLPFC and OFC than MDNSA patients during the threat stimuli. MDSA patients with higher impulsivity and more depressive symptoms had greater activation in the DLPFC to threat stimuli, and the patients with greater hopelessness and more depressive symptoms had greater OFC activation to the threat stimuli. We did not witness these trends in healthy individuals, although the activation of OFC and DLPFC during the task was similar between MDSA patients and healthy individuals, which means that the patients had different pathophysiology of the brain activation during the negative emotional stimuli from the healthy subjects. For the MDNSA patients, weak activation in the left DLPFC and left OFC to threat stimuli was observed, compared the MDSA patients and healthy subjects. The MDNSA patients demonstrated that higher hopelessness and depression was not associated with the OFC or DLPFC activation, and normal impulsivity related to weak activation in the both regions (Table 2). The results suggest that the activation of OFC and DLPFC to negative emotional cognitive stimuli, personality traits is associated with suicidal behavior, indicating the findings are involved in the pathophysiology of suicidality in mood disorders.

**Table 2:** Summary of results.

	Negative emotional cognitive stimuli	Correlation with impulsivity	Correlation with depression or hopelessness
MDSA	DLPFC ↑	DLPFC	DLPFC
	OFC ↑		OFC
MDNSA	DLPFC ↓	DLPFC	n.s.
	OFC ↓	OFC	
Healthy	DLPFC ↑	n.s.	n.s.
	OFC ↑		

*MDSA* mood disorder patients with a history of suicide attempt, *MDNSA* mood disorder patients with no history of suicide attempt, *DLPFC* dorsolateral prefrontal cortex, *OFC* orbitofrontal cortex, *n.s.* not significant.

### The DLPFC and SRB

The DLPFC facilitates decision-making and plays a crucial role in decision-making conflict^45^. This region also forms a network with the dorsomedial prefrontal cortex and parietal region to regulate thoughts, emotions, and behavior^46^. Several studies on SRB have reported a decreased volume of the DLPFC in MDSA^22,47^. Other studies have shown that adolescent MDSA had higher activation in areas (including the bilateral DLPFC) than MDNSA^48^, and that patients with mood disorders with psychotic features and high DLPFC activity during cognitive control tasks exhibited SRB^49^. Previous studies partially endorse our results. Nevertheless, some studies do not support our finding that the activity of the DLPFC is reduced in MDSA when evaluating risky versus safe choices^50^.

In a suicide-related study using NIRS, depressed patients with suicidal ideation displayed less activity in the right DLPFC than depressed patients without suicidal ideation using the VFT task^51^, which is different from our results. A previous study used the VFT of a neuropsychological task, which examines fluent cognition in the brain, and a simple assessment of suicidal ideation using one item for the HAMD score; in contrast, we employed flexible emotional cognition and a scale from the C-SSRS that rigorously assess suicide attempts and suicidal ideation. The different tasks and assessments of suicide attempts may be associated with different outcomes between past and current NIRS studies. Given the importance of defining SRB in suicide research^52^, our study design is rigorous in terms of scrutinizing the link between emotions, brain activity, and suicide-relatedness, which may reflect a more accurate pathophysiology of the brain in terms of risky behaviors in persons with mood disorders.

### The OFC and SRB

The OFC regulates the output of the amygdala and is involved in decision-making and emotional processing^45,53^. A meta-analysis of neuropsychological studies found that impaired decision-making occurs in MDSA^54^. Evidence of an association between mood disorders and the OFC has been accumulated in many neuroimaging studies^55,56^. Research has also shown an association between SRB and the OFC; for example, structural imaging studies have reported that patients with depression and a history of suicide attempts have smaller gray matter volume in the left OFC compared to healthy individuals^57^, and adolescents and young adult patients with BD who have a history of suicide attempts have smaller gray matter volume in the lateral OFC compared to healthy individuals^58^.

In functional imaging studies, veterans with suicidal ideation exhibited high activity in the lateral OFC during false responses on inhibitory control tasks; the findings imply that functional abnormalities in the OFC are a risk factor for suicide attempts, which partially supports our results^59^. In a suicide-related study of depression using NIRS, lower OFC activity during a VFT was observed in MDSA compared to MDNSA^25^, and in patients who experienced suicidal ideation versus those who did not^51^. These outcomes differ from ours, but as noted in the section on the DLPFC, it is possible that differences in the tasks and rigor of the assessment of suicidal ideation and suicide attempts may have led to differences in results.

### The relationships among the DLPFC, OFC, and personality traits

In the MDSA, activity in the DLPFC region was associated with impulsivity and depressive symptoms, while activity in the OFC is associated with hopelessness and depressive symptoms. In contrast, MDNSA patients were associated with the DLPFC and OFC regions and impulsivity, but not with other psychiatric symptoms or traits. Disruption of the combination of the DLPFC and inferior frontal gyrus regions (which control cognition) and the OFC and ventral anterior cingulate gyrus regions (which are associated with low self-esteem and future pessimism) may lead to SRB^22^. In an fMRI study using a decision-making task with emotional facial feedback, MDSA and MDNSA displayed different activations of the OFC and DLPFC^60^. Another fMRI study using a similar decision-making task demonstrated that MDSA patients showed higher activation of the OFC and DLPFC during a financial win compared to MDNSA, suggesting that these regions are associated with the impairment of decision-making in MDSA^50^.

The relationship between SRB and impulsivity in mood disorders has been reported in several studies^61,62^. Decreased serotonin 2A receptor binding in the DLPFC is associated with impulsivity and aggression in suicidal patients^63^, and structural abnormalities in the DLPFC and serotonergic nervous system are involved in SRB^22^. Furthermore, patients with BD and higher impulsivity had smaller brain volumes in the OFC^64^. Hopelessness and impulsivity are also included as suicide risk factors in patients with MDD^65^. Depressed patients in an NIRS study who self-rated depression severities worse than observer-rated depression severities had higher hopelessness and a history of suicide attempts, which was related to abnormal DLPFC function during the VFT^66,67^.

**Figure 4:**
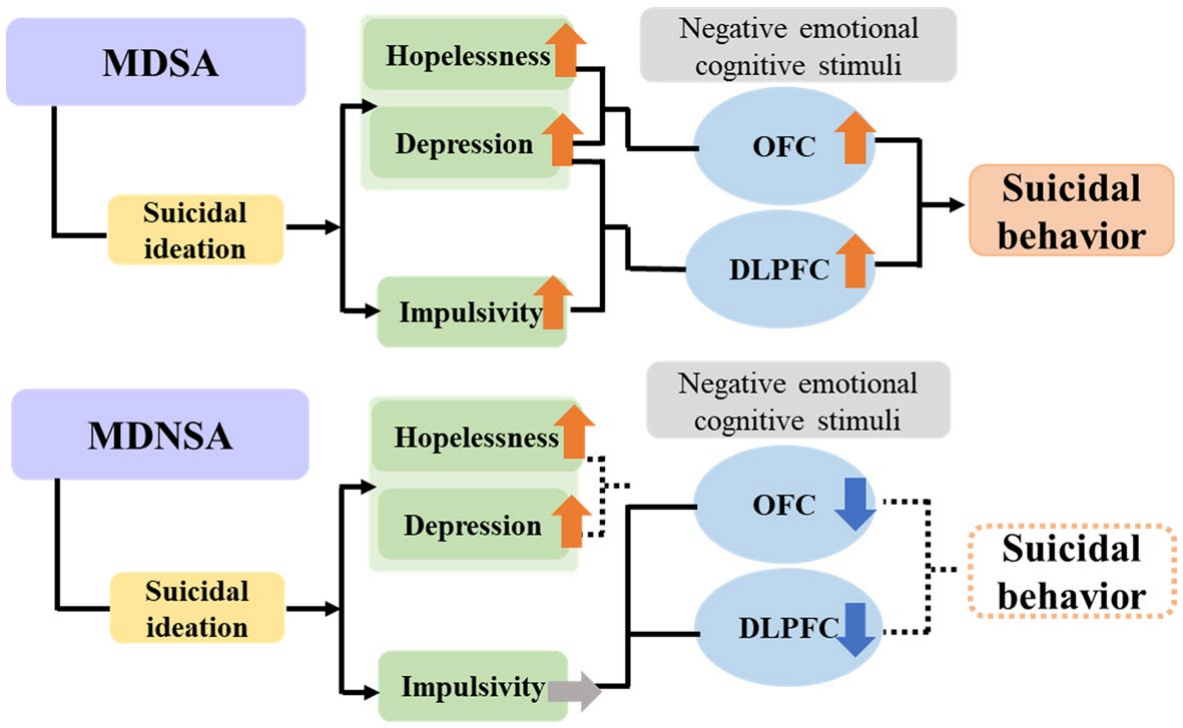
A model of frontal activity in mood disorders, including personality traits and SRB. We have modified the figure of Mann et al.^4^ based on our results.

Mann et al. proposed a stress-diathesis model of suicidal behavior^4^. This model states that stressors include the aggravation of mental illness and psychosocial burden, which in turn lead to suicidal ideation. Suicidal ideation has been added to diathesis factors in that serotonergic and noradrenergic dysfunction exacerbates hopelessness and impulsivity, finally leading to suicide attempts. Together with our results, we considered a model that includes brain dysfunction (Fig. 4). In this model, suicidal ideation arises as the condition worsens in MDSA. In addition to worsening depressive symptoms, the diathesis personality traits of high impulsivity and strong feelings of hopelessness are associated with an overreaction of the OFC and DLPFC during negative emotional cognitive stimuli, ultimately leading to SRB. On the other hand, in the patient group, this pathway was interrupted midway; that is, even when suicidal ideation occurred, the diathesis factors included high hopelessness but not impulsivity, and the OFC and DLPFC had reduced activity due to negative emotional cognitive stimuli, which were only associated with their low brain activity and normal impulsivity. As a result, this pathway is fragmented and does not lead to SRB. Clearly, this is speculation, and an accumulation of evidence—including the confirmation of reproducibility—is required to verify this speculation.

### Limitations

Our study has several limitations. First, NIRS, by nature, has a lower spatial resolution than fMRI. Second, NIRS can only measure the brain’s surface. Therefore, it cannot directly measure the function of the anterior cingulate gyrus, amygdala, or hippocampus, which are deep areas of the brain involved in impulsivity and SRB^22^. Hence, we cannot rule out the possibility that the activity of these unmeasured brain regions may have affected our results. Third, the sample size was insufficient. Fourth, the patients included a mix of persons with MDD and BD, including depressed and partially remitted patients. Although the proportions of these two conditions did not differ between the two groups, and some studies point to similarities rather than heterogeneity in brain activity between MDD and BD^68^, we cannot rule out the possibility that heterogeneities may have influenced the outcomes. Fifth, most patients were taking psychotropic medications during the study, and it is possible that the medication had an effect on the NIRS signal. To our knowledge, there is no clear evidence that drugs affect NIRS signals, and there are reports of no difference in NIRS brain activity measured by VFT before and after antidepressant or antipsychotic medication in patients with MDD^69^ and schizophrenia^70^. In addition to these studies, the fact that there were no significant differences in the medications taken by the MDSA and MDNSA patients suggests that the impact of medications on NIRS-related findings cannot be completely ruled out. However, if present, the effect is likely to be subtle. We are presently waiting for more evidence to accumulate regarding the relationship between drugs and NIRS signals.

## Conclusion

In this study, we evaluated brain activity in the frontotemporal regions during an emotion recognition task in MDSA and MDNSA patients and healthy individuals using NIRS. The results suggest that the activation of OFC and DLPFC to negative emotional cognitive stimuli, personality traits is associated with suicidal behavior, indicating the findings are involved in the pathophysiology of suicidality in mood disorders. Future research will need to elucidate the activation of other brain regions on different emotional cognitive and other personality traits in mood disorder patients with suicide risks to better understand suicidal brain pathophysiology and prevent suicidal behaviors.

## Data Availability

The datasets generated and/or analyzed during the current study are available from the corresponding author upon reasonable request.
